# Increased pupillary constriction velocity in benign essential blepharospasm associated with photophobia

**DOI:** 10.1371/journal.pone.0217924

**Published:** 2019-06-04

**Authors:** Yong-Soo Byun, Sung-Eun Kim, Ji-Sun Paik, Suk-Woo Yang

**Affiliations:** Department of Ophthalmology and Visual Science, Seoul St. Mary’s Hospital, College of Medicine, The Catholic University of Korea, Seoul, Republic of Korea; Roskamp Institute, UNITED STATES

## Abstract

We evaluated whether the pupillary light reflex is altered in benign essential blepharospasm patients. Twenty-three patients with benign essential blepharospasm, 47 with reflex blepharospasm, and 29 dry eye disease controls were included. Pupillary light reflex-related parameters were measured under mesopic (10 lux) and photopic illuminance (200 lux) using an infrared pupillometer. Additionally, we assessed photophobia grade, eyelid function, and dry eye disease-related parameters. There were no differences in age, sex predominance, or dry eye disease-related parameters among the three groups, or in photophobia grade and eyelid function between benign essential blepharospasm and reflex groups. Constriction velocity and maximum constriction velocity in the mesopic condition were significantly greater in the benign essential blepharospasm group (3.26 ± 0.56 and 5.27 ± 0.90 mm/s) than in reflex (2.86 ± 0.62 and 4.59 ± 1.00 mm/s) or dry eye disease groups (2.96 ± 0.46 and 4.72 ± 0.67 mm/s). Constriction velocity and maximum constriction velocity in the mesopic condition positively correlated with photophobia grade (*r* = 0.525 and 0.617, *P* = 0.025 and 0.006) in the benign essential blepharospasm group. Pupillary light reflex may be related to the pathophysiology of benign essential blepharospasm with photophobia. Further studies are required to reveal connections among pupillary light reflex, photophobia, and focal dystonia in benign essential blepharospasm patients with photophobia.

## Introduction

Benign Essential Blepharospasm (BEB) is a form of focal dystonia that manifests as involuntary bilateral contraction or spasm of the eyelids without neurological causes. It is predominant in females and mostly affects people over 50 years old.[1] Starting with mild twitches, it usually progresses to excessive blinking, forceful bilateral spasm of eyelids, and in advanced cases, to involuntary eye closure or functional blindness.[2] Its etiologies are unclear and multifactorial. BEB is clinically diagnosed by exclusion diagnosis and must be distinguished from secondary blepharospasm, which can occur in association with underlying neurologic diseases, or a specific ocular disease such as in reflex blepharospasm secondary to ocular irritation.[3] Research using animal models has proposed that possible pathophysiologic mechanisms of BEB may include basal ganglia dysfunction, ion channelopathy, sensitization of the trigeminal system, and a predisposing genetic background.[1, 4]

Although there is no consensus regarding its definition, photophobia is generally referred to as a sensory response in which light causes ocular pain, periocular pain, or headache; otherwise, it may cause avoidance behavior without manifest pain.[5] According to previous studies, photophobia is present in up to 94% of BEB patients.[6–8] In BEB, photophobia may be the non-motor phenomenon of disorder or result from comorbid ocular diseases such as dry eye disease, blepharitis, and intraocular inflammation. Although the pathophysiological mechanism of photophobia remains elusive, several studies have suggested that intrinsic photosensitive retinal ganglionic cells (ipRGCs), a subtype of retinal photoreceptors, play a critical role in photophobia.[9–11] ipRGCs are well-known to contribute to non-image-forming visual functions, such as circadian rhythm and pupillary light reflex.[12] These cells send excitatory signals to the Edinger-Westphal nucleus via the olivary pretectal nucleus, which controls pupillary constriction,[13] rather than to the primary visual cortex via the lateral geniculate nucleus.

Therefore, we expected that pupillary light reflex may be altered in BEB patients with photophobia, because ipRGCs are involved in both photophobia and pupillary light reflex. In this study, we assessed the pupillary light reflex by using a pupillometer in the BEB, reflex blepharospasm, and the dry eye disease (DED; control) groups to determine whether pupillary light reflex is altered in conjunction with photophobia in BEB patients.

## Materials and methods

### Subjects

This cross-sectional observational study was conducted at St. Mary’s Hospital, Catholic University of Korea with the approval of Institutional Review Board/Ethics Committee of St. Mary’s Hospital (KC17RCSI0644). All patients provided informed consent after being briefed about the purpose of the study and tests. The study protocol complied with the tenets of the Declaration of Helsinki. Twenty-three patients with BEB (BEB group) and 47 patients with reflex blepharospasm (reflex group) caused by DED were included. The diagnoses of BEB and reflex blepharospasm were made following complete neurological and ophthalmological examinations. Anesthetic eyedrops (Alcaine, proparacaine hydrochloride 0.5%, Alcon Laboratories, Inc., Fort Worth, TX, USA) were used to distinguish the two types of blepharospasm in cases that could not be diagnosed solely on the basis of clinical features and past history. If the blepharospastic condition improves with anesthetic-induced ocular numbness, BEB is less likely to occur. All patients with blepharospasm were those who visited the hospital for regular injection of botulinum toxin after the drug effect had ended, or those who had no previous history of botulinum toxin injection. The control group consisted of 29 DED patients without signs of dystonia, such as excessive or abnormal blinking. The exclusion criteria were as follows: any ocular surgeries within 1 year; any optical abnormalities that could cause light scattering; acute inflammatory conditions including conjunctivitis, keratitis, or uveitis; retinal or optic disc abnormalities strongly suspected to cause photophobia; and topical or systemic medications that affect the pupillary response.

### Clinical assessment and pupillary measurement

DED was diagnosed if typical symptoms and at least one sign of low Schirmer I value (< 10 mm), reduced tear breakup time (TBUT < 10 sec) and positive staining were present.[14] The DED-related parameters including TBUT,[15] Schirmer I test score (with anesthesia), corneal staining score (0 to 3),[16] and the ocular surface disease index (OSDI) questionnaire[17] were evaluated. The photophobia grade was evaluated according to the scale previously reported[18]: Grade 0, no photophobia or discomfort; Grade 1, slight difficulty with light causing occasional eye blinking; Grade 2, slight difficulty with light causing regular eye blinking; Grade 3, moderate difficulty with light requiring the wearing of sunglasses; Grade 4, severe difficulty with light requiring the wearing of sunglasses in a quasipermanent manner; Grade 5, extreme difficulty with light requiring the patient to remain inside and an inability to bear natural light even with sunglasses.

Eyelid function was assessed using the modified grading scheme for the severity of spasm (0 = no spasm; 1 = mild spasm at stimulation only; 2 = visible spasm without impairment of daily life; 3 = visible spasm with impairment of daily life; 4 = severe spasm with impairment of daily life), eyelid closing force (1 = flaccid; 2 = overcome with minimum resistance; 3 = overcome with moderate resistance; 4 = normal strength), and functional visual status (1 = functional blindness; 2 = dependent: unable to go out alone; 3 = poor function: unable to watch TV, read or drive; 4 = moderate function: unable to read but able to work; 5 = inconvenience: intermittent discomfort but able to drive and work; 6 = normal) by oculoplastic specialists (S-. W. Y., H-. J. Y., S-. E. K.).[19]

Pupillary light reflex was examined using an infrared pupilometer (NPi-100, NeurOptics Inc. Irvine, CA, USA). A pupilometer was placed on each eye after 5-minute adaptation in a dark room with mesopic illumination (approximately 10 lux). The dynamic change of the pupil was detected after the integrated LED light flashed. The same procedure was repeated in a lighted room (approximately 200 lux). The device automatically calculated the maximum diameter (MAX), minimum diameter (MIN), %change of pupil diameter (%CH), latency of constriction (LAT), constriction velocity (CV), maximum CV (MCV), and dilation velocity (DV) of the pupil during the pupillary light reflex.

### Statistical analysis

We analyzed data from one eye of each patient (the eye in which the pupillary light reflex was first measured). The normality of the data was determined using the D'Agostino–Pearson omnibus test. Then, the Mann–Whitney U test was used to compare the photophobia grade and lid function-related parameters between the BEB group and reflex group; the Kruskal–Wallis one-way analysis of variance with Dunn’s post hoc comparison test was used to compare DED-related parameters and pupillary light reflex-related parameters among three groups. The Spearman correlation test was used to analyze correlations between photophobia grade and pupillary light reflex-related parameters. Statistical analyses and graph construction were conducted by using GraphPad Prism 5 (GraphPad software, San Diego, CA, USA). A two-sided *P*-value < 0.05 was considered statistically significant.

## Results

There were no differences in age and sex predominance among the BEB, reflex, and DED groups; all patients of the BEB and reflex groups exhibited photophobia and dry eye symptoms (Table 1).

**Table 1:** Demographic characteristics of patients with benign essential blepharospasm and reflex blepharospasm and dry eye disease control.

	BEB group	Reflex group	DED group	*P* value ^a^
**Number of patients**	23	47	29	-
**Age (mean ± SD)**	66.26 ± 9.19	66.70 ± 9.07	61.79 ± 7.34	0.0902
**Female (number, [%])**	20 (87.0%)	43 (91.5%)	25 (86.2%)	-
**Dry eye symptoms (number, [%])**	23 (100%)	47 (100%)	29 (100%)	-
**Photophobia (number, [%])**	23 (100%)	47 (100%)	0 (0%)	

BEB, benign essential blepharospasm; DED, dry eye disease; SD, standard deviation

^a^ *P* value was calculated using the Kruskal–Wallis one-way analysis of variance test

There were no differences in eyelid functions (severity of spasm, eyelid closing force, or functional visual status) or photophobia grade between the BEB and reflex groups, whereas, of the DED-related parameters, only OSDI score showed a significant difference among the three groups (Table 2). Post hoc comparisons with Dunn’s test revealed that the OSDI score was significantly higher in the BEB and reflex groups than in the DED control (*P* < 0.05 for both).

**Table 2:** The lid function, dry eye disease-related parameters, and photophobia grade in patients with benign essential blepharospasm and reflex blepharospasm.

	Mean ± SD (range)			*P* value
	BEB group	Reflex group	DED group	
**Photophobia grade**	2.09 ± 1.24 (1 to 4)	2.62 ± 0.90 (1 to 4)	-	0.4757^a^
**Lid function**				
**Severity of spasm**	2.79 ± 0.97 (1 to 4)	2.62 ± 0.90 (1 to 4)	-	0.4757^a^
**Eyelid closing force**	3.56 ± 0.89 (1 to 4)	3.64 ± 0.56 (2 to 4)	-	0.8071^a^
**Functional visual status**	3.87 ± 1.13 (2 to 5)	3.88 ± 0.99 (2 to 5)	-	0.9878^a^
**DED-related parameters**				
**TBUT (sec)**	5.22 ± 2.68 (0 to 10)	5.94 ± 2.36 (2 to 10)	5.00 ± 1.78 (2 to 8)	0.1904^b^
**Schirmer I value**	6.30 ± 3.10 (2 to 15)	5.81 ± 2.75 (2 to 15)	5.90 ± 3.21 (2 to 15)	0.7091^b^
**Corneal staining score**	0.44 ± 0.84 (0 to 3)	0.55 ± 0.72 (0 to 3)	0.77 ± 0.82 (0 to 3)	0.1502^b^
**OSDI score**	37.4 ± 20.6 (2.1 to 77.3)	45.4 ± 18.7 (2.8 to 83.3)	24.0 ± 13.2 (2.1 to 55.5)	0.0001^c^

SD, standard deviation; BEB, benign essential blepharospasm; DED, dry eye disease; TBUT, tear breakup time; MGD, Meibomian gland dysfunction; MG, Meibomian gland; OSDI, ocular surface disease index

^a^*P* values were calculated by the Mann-Whitney U test between two groups

^b^*P* values were analyzed by the Kruskal–Wallis one-way analysis of variance among three groups

^c^*P* value was calculated by the Kruskal–Wallis one-way analysis of variance among three groups. Post hoc comparison with Dunn’s test showed significant differences between the DED and other groups.

Of the pupillary light reflex-related parameters, CV and MCV measured under mesopic illuminance significantly differed among the three groups (*P* = 0.0047 and 0.0027, respectively, Table 3). Mesopic CV and MCV in the BEB group (3.26 ± 0.56 and 5.27 ± 0.90 mm/s) were significantly greater than in the reflex group (2.86 ± 0.62 and 4.59 ± 1.00 mm/s) or the DED control (2.96 ± 0.46 and 4.72 ± 0.67 mm/s) by Dunn’s post hoc comparison (*P* < 0.05 for both).

**Table 3:** The pupillary light reflex-related parameters in patients with benign essential blepharospasm and reflex blepharospasm.

Illuminance	Parameters	Mean ± SD (range)			*P* value
		BEB group	Reflex group	DED group	
**Mesopic**	**MAX (mm)**	5.08 ± 0.76 (3.56 to 6.52)	4.79 ± 0.65 (3.58 to 6.49)	4.96 ± 0.73 (3.49 to 6.34)	0.2172
	**MIN (mm)**	3.00 ± 0.47 (2.24 to 3.62)	2.96 ± 0.50 (2.00 to 4.27)	2.88 ± 0.49 (2.07 to 3.93)	0.5058
	**%CH**	40.78 ± 4.70 (33.0 to 49.0)	38.15 ± 6.90 (13.0 to 47.0)	41.24 ± 5.47 (27.0 to 48.0)	0.0707
	**LAT (s)**	0.24 ± 0.03 (0.15 to 0.28)	0.25 ± 0.03 (0.19 to 0.31)	0.24 ± 0.03 (0.19 to 0.31)	0.1912
	**CV (mm/s)**	3.26 ± 0.56 (1.83 to 4.09)	2.86 ± 0.62 (1.10 to 3.94)	2.96 ± 0.46 (1.87 to 3.90)	0.0047^**a**^
	**MCV (mm/s)**	5.27 ± 0.90 (3.09 to 6.47)	4.59 ± 1.00 (1.79 to 7.15)	4.72 ± 0.67 (3.20 to 5.85)	0.0027^**a**^
	**DV (mm/s)**	1.26 ± 0.22 (0.72 to 1.54)	1.18 ± 0.26 (0.64 to 2.10)	1.17 ± 0.27 (0.14 to 1.50)	0.0941
**Photopic**	**MAX (mm)**	3.47 ± 0.76 (2.00 to 4.93)	3.34 ± 0.58 (2.01 to 4.40)	3.15 ± 0.48 (2.38 to 4.07)	0.1672
	**MIN (mm)**	2.55 ± 0.49 (1.53 to 3.37)	2.48 ± 0.42 (1.62 to 3.46)	2.33 ± 0.31 (1.82 to 2.97)	0.1293
	**%CH**	26.04 ± 7.07 (7.0 to 37.0)	25.33 ± 5.62 (14.0 to 36.0)	25.48 ± 5.42 (13.0 to 37.0)	0.7457
	**LAT (s)**	0.25 ± 0.05 (0.12 to 0.37)	0.25 ± 0.03 (0.19 to 0.31)	0.25 ± 0.03 (0.19 to 0.28)	0.8466
	**CV (mm/s)**	1.80 ± 0.67 (0.58 to 2.87)	1.77 ± 0.61 (0.52 to 3.58)	1.80 ± 0.59 (0.68 to 3.27)	0.9618
	**MCV (mm/s)**	2.83 ± 1.02 (1.06 to 4.68)	2.66 ± 0.84 (0.90 to 4.84)	2.61 ± 0.81 (0.90 to 4.63)	0.6554
	**DV (mm/s)**	0.97 ± 0.29 (0.37 to 1.53)	0.96 ± 0.30 (0.33 to 1.53)	0.97 ± 0.29 (0.39 to 1.73)	0.9591

SD, standard deviation; BEB, benign essential blepharospasm; DED, dry eye disease; MAX, maximum diameter; MIN, minimum diameter; %CH, %change of pupil diameter; LAT, latency of constriction; CV, constriction velocity; MCV, maximum constriction velocity; DV, dilation velocity

^a^Post hoc comparison with Dunn’s test showed significant differences between the benign essential blepharospasm group and other groups.

**Fig 1:**
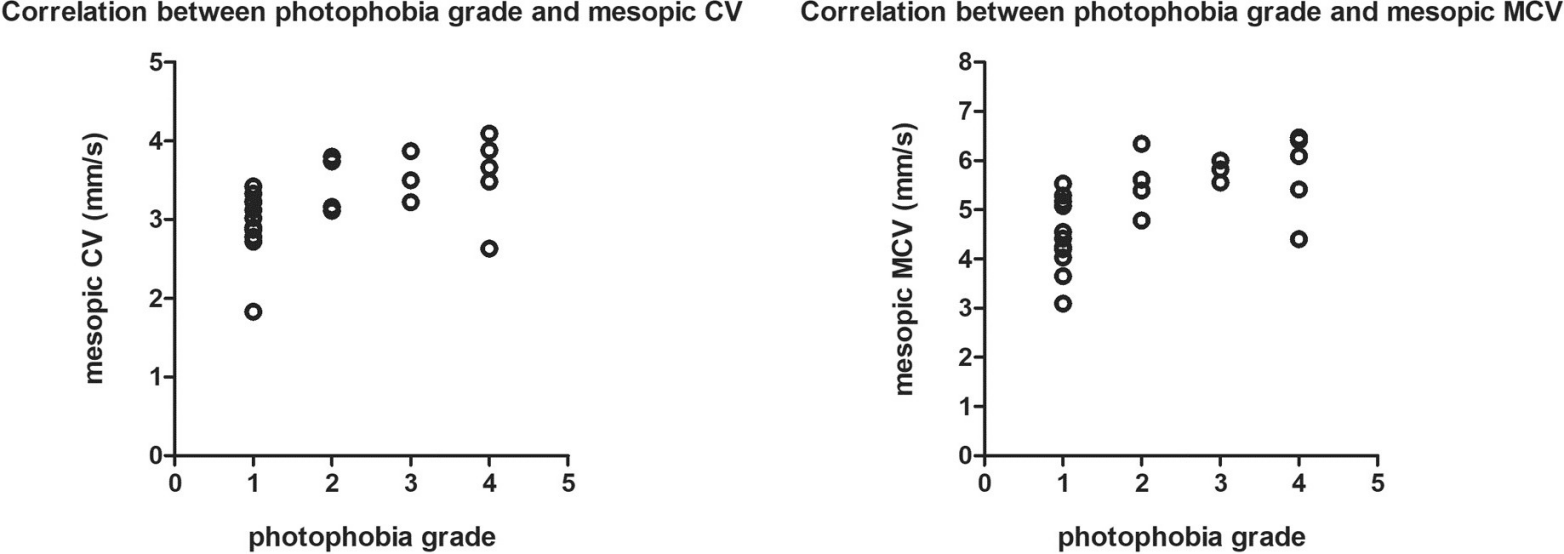
Scatter graphs illustrating the correlations between photophobia grade and both mesopic constriction velocity (CV) and maximum constriction velocity (MCV) in patients with benign essential blepharospasm.

Additionally, mesopic CV and MCV significantly correlated with photophobia grade in the BEB group (*r* = 0.525 and 0.617, *P* = 0.025 and 0.006, respectively; Table 4). Fig 1 demonstrates the correlation between these values and photophobia grade in the BEB group.

**Table 4:** The spearman’s correlation coefficients between the photophobia grade and the pupillary light reflex-related parameters in 23 patients with benign essential blepharospasm.

Illuminance	Parameters	*Rho*^*a*^	*P* value
**Mesopic**	**MAX (mm)**	0.161	0.523
	**MIN (mm)**	0.045	0.858
	**%CH**	0.424	0.080
	**LAT (s)**	-0.073	0.774
	**CV (mm/s)**	0.525	0.025
	**MCV (mm/s)**	0.617	0.006
	**DV (mm/s)**	0.385	0.114
**Photopic**	**MAX (mm)**	-0.079	0.614
	**MIN (mm)**	-0.173	0.268
	**%CH**	0.232	0.134
	**LAT (s)**	-0.152	0.329
	**CV (mm/s)**	-0.023	0.882
	**MCV (mm/s)**	0.008	0.961
	**DV (mm/s)**	0.093	0.554

MAX, maximum diameter; MIN, minimum diameter; %CH, %change of pupil diameter; LAT, latency of constriction; CV, constriction velocity; MCV, maximum constriction velocity; DV, dilation velocity

^a^Spearman rank correlation coefficient.

## Discussion

Our study showed that pupillary CV and MCV under mesopic illuminance were significantly greater in the BEB group than in the reflex group and the DED control, and that these parameters positively correlated with photophobia grade in the BEB group. Thus far, there has been no study regarding pupillary responses and photophobia in BEB patients. These results suggest the presence of alterations related to the pathway of pupillary light reflex in patients who exhibit BEB with photophobia.

First, the alterations may comprise increased activity of ipRGCs, which are a major source of retinal input to pupillary light reflex pathway[13]; thus, increased activity of these cells may lead to an increased pupillary constriction response. ipRGCs directly or indirectly connect to thalamic nuclei, which are associated with somatic sensation and pain.[11] Therefore, it is reasonable to speculate that increased activity of ipRGCs may be related to greater photophobia. Additionally, susceptibility to short wavelength light has been demonstrated, both in the pupillary light reflex and photophobia. It is known that the pupillary constriction response to blue light is greater than the response to red light.[20] Patients with BEB are more susceptible to short-wavelength light; the FL-41 chromatic tint, which blocks short-wavelength light transmission, reduced blinking frequency and photophobia in these patients.[21] These findings are consistent with preferential activation of ipRGCs by short-wavelength light, because these cells have a peak spectral sensitivity at approximately 480 nm wavelength.[13]

Second, the parasympathetic efferent arm of pupillary light reflex pathway projecting to iris sphincter muscle may be involved in greater pupillary CV in patients that exhibit BEB with photophobia. McCann et al.[22] reported that 13 of 19 BEB patients showed improvement in eyelid spasm, ocular irritation, and photophobia after cervical sympathetic blockade; they speculated that BEB may be a form of sympathetically maintained pain syndrome, more recently termed complex regional pain syndrome (CRPS) type I. Although the pathogenic mechanism of CRPS has not been fully elucidated, its main cause may be inappropriate crosstalk between sensory and motor fibers at the affected site.[23] In contrast to previous speculation (indicated by the name), sympathetic tone is reduced in the affected region, although sympatho-afferent coupling may occur.[23] The iris may be a site where inappropriate sympatho-afferent coupling occurs because of its abundant muscles, vessels, autonomic efferent nerves, and sensory afferent nerves. In this case, the parasympathetic-driven pupillary constriction is suspected to oppose the weakened sympathetic pupil dilation system, resulting in greater pupillary CV. Therefore, the pathogenic mechanism of CRPS may partly explain the correlation between pupillary CV and photophobia grade in BEB.

In contrast to mesopic illuminance, CV and MCV under photopic illuminance did not show intergroup differences. It is difficult to explain the disparity in findings between mesopic and photopic conditions, because no studies have been reported regarding background illumination intensity and the pupillary light reflex. Therefore, additional efforts should be made to develop a standard method for assessment of dynamic and static pupillary responses, in order to clarify the relationship between pupillary light reflex and photophobia in BEB patients.

In this study, the BEB and reflex groups showed no differences in TBUT, Schirmer’s test, or corneal staining scores, compared with the DED group; however, the OSDI (indicative of subjective symptoms) was significantly higher in the BEB and reflex groups than in the DED group. This suggests that the ocular discomfort was more severe than observed DED-related signs in blepharospasm patients with DED, and that blinking abnormality or eyelid spasm itself contributed significantly to subjective symptoms in these patients. Additionally, the BEB and reflex groups showed no differences in photophobia grade, as well as DED-related parameters and eyelid functions, which can both affect and be affected by photophobia. A previous study by Grandas and colleagues reported that 55.3% of patients complained of ocular irritation before or at the onset of BEB.[24] Inadequate blinking can also deteriorate tear production, distribution, drainage, and ocular surface inflammation in patients with BEB.[25, 26] Therefore, clinical indicators of DED and sensory symptoms are not beneficial in discriminating BEB and reflex blepharospasm. Instead, characteristic features of eyelid dystonia and careful history taking are important to discriminate between BEB and reflex blepharospasm; the development of additional diagnostic tools will be beneficial for differential diagnosis of BEB. In this respect, measurement of the pupillary constriction response may facilitate differentiation of patients with BEB from patients with photophobia and traits of dystonia.

The present study had several limitations. We assessed the severity of eyelid spasm using a simple grading system; we could not exclude the clinical heterogeneity of BEB patients based on traits of dystonia. Recent studies have emphasized possible pathophysiological differences based on clinical features of orbicularis oculi muscle spasms.[27, 28] Additionally, some variables could be affected by botulinum toxin (BoNT) treatment, which is known to modulate blinking rate; its effect may depend on the clinical heterogeneity of BEB.[29] However, all patients in the BEB and reflex groups in this study either had no previous BoNT treatment, or had visited for additional BoNT injection after the drug effect had ended. The proportions of patients who had undergone treatment with botulinum toxin (BoNT) were 73.9% (17 of 23) and 63.8% (30 of 47) in the BEB and reflex groups; these proportions were not significantly different (*P* = 0.4023 by Chi-squared test). Therefore, we suspect that the BoNT factor might have minimal effect on the variables in this study. In further studies, the detailed assessment of the traits of dystonia, such as blinking frequency, duration, and force, as well as phenotypic changes over time and information regarding BoNT treatment, should be considered to discern possible connections between pupillary light reflex and photophobia, as well as blinking abnormality in BEB.

In conclusion, our study revealed that BEB patients with photophobia showed a greater pupillary constriction response in the mesopic condition, which positively correlated with the subjective severity of photophobia. The pupillary constriction response may be of clinical significance as a manifest response reflecting sensory phenomenon in BEB with photophobia, although it is not known whether these results are pathogenic consequences of BEB or are suggestive of the involvement of the pupillary constriction response in BEB pathogenesis. In the future, the increased activity of the pupillary light reflex pathway, which is a possible mechanism for these findings, should be clarified through additional studies.
